# Negative Regulation of GADD34 on Myofibroblasts during Cutaneous Wound Healing

**DOI:** 10.1155/2014/137049

**Published:** 2014-08-19

**Authors:** Lintao Liu, Naomi Nishio, Sachiko Ito, Yuriko Tanaka, Ken-ichi Isobe

**Affiliations:** Department of Immunology, Nagoya University Graduate School of Medicine, 65 Turumai-cho, Showa-ku, Nagoya, Aichi 466-8550, Japan

## Abstract

The growth arrest and DNA damage-inducible protein, GADD34, has been proved to be involved in TGF-*β* signaling pathway and correlates with cell death, which are two important mechanisms in regulating myofibroblast differentiation and apoptosis during tissue repair. But roles of GADD34 in myofibroblasts differentiation and apoptosis remain unknown. To investigate the function of GADD34 in these processes, we subjected WT and GADD34^−/−^ mice to dermal wound healing. Here we show that GADD34^−/−^ mice exhibited accelerated wound closure compared with WT mice. In addition, GADD34^−/−^ mice showed increased number of myofibroblasts, elevated collagen production, and decreased cell apoptosis during wound healing. Moreover, we found that GADD34^−/−^ mice showed increased phosphorylation of Smad3 and lower level of cleaved caspase-3. Thus these results indicate that GADD34 appears to suppress myofibroblast differentiation through inhibiting Smad3-dependent TGF*β* signal pathway and promote its apoptosis by activating caspase-3 pathway.

## 1. Introduction

Cutaneous wound healing process depends on three major phases—inflammation, tissue formation, and tissue remodeling [1]. Among them tissue formation begins with reepithelialization of wounds by epidermal cells migrating from skin appendages such as hair follicles. Next, myofibroblasts, a subgroup of cells with an expression of *α*-smooth muscle actin (*α*-SMA) within cytoplasmic stress fibers, proliferate and are recruited to wound site. One of the most important functions of myofibroblasts is to generate contractile forces to shrink wound size by deposition of collagen-rich extracellular matrix, including collagen type I, collagen type III, fibronectin, and proteoglycan [2–5]. As the wound becomes epithelialized and the scar forms, myofibroblasts undergo apoptosis and disappear from granulation tissue, although this mechanism has not been entirely understood until now [6].

Differentiation of myofibroblasts during wound healing is mainly triggered by transforming growth factor *β*1 (TGF-*β*1) through Smad signaling. Due to ligand binding, the signal transduction is initiated with the formation of heterotetrameric active receptor complexes of TGF-*β* type I receptor (TGF-*β*1RI) and TGF-*β* type II receptor (TGF-*β*1RII), which in turn results in phosphorylation of TGF-*β*1RI by TGF-*β*1RII. TGF-*β*1RI induces subsequent phosphorylation and heterodimerization of Smad2 and Smad3 (receptor-regulated Smads, R-Smads). The phosphorylated Smad2/3 complexes associate with the common-mediator Smad4 (Co-Smad), which accumulate in the nucleus to regulate gene transcription in concert with other transcription factors. This pathway is regulated negatively by inhibitory Smads such as Smad7, which inactivates TGF-*β*1RI to prevent phosphorylation of R-Smads [7, 8].

GADD34, as a member of growth arrest and DNA damage-inducible protein family, is upregulated in response to a variety of cellular stresses, including endoplasmic reticulum stresses [9], nutrient deprivation [10], oxidative stress [11], heat shock [12], and energy depletion [13]. Expression of GADD34 is correlated with apoptosis [14, 15] and its overexpression facilitates apoptosis associated with ionizing radiation [16]. Recently, GADD34 has been shown to be involved in TGF*β* signal pathway by cooperating with Smad7 to dephosphorylate TGF*β* type I receptor, which plays a crucial role in myofibroblast differentiation [17]. The observed association of GADD34 with cellular apoptosis and TGF*β* signal pathway promoted us to ask whether GADD34 might participate in myofibroblast differentiation and apoptosis during wound healing.

## 2. Materials and Methods

### 2.1. Animal Experiments

10-week-old WT (C57BL/6) male mice were purchased from SLC (Sizuoka, Japan). GADD34-deficient (GADD34^−/−^, KO) mice were generated as previously described [18]. Originally, GADD34^−/−^ mice were produced from ES cells with a C57BL/6 and 129 background. These mice were backcrossed to WT for up to nine generations. Mice were maintained in the Animal Research Facility at the Nagoya University Graduate School of Medicine under specific pathogen-free conditions and were used according to institutional guidelines.

For wound healing studies, mice were anesthetized by intraperitoneal injection of avertin (250 uL/20 g mice) and then a full-thickness 3 mm skin wound was made by punch biopsy onto the middle back skin of 10-week-old adult male mice. For each time point examined, wound tissue biopsies were collected with 6 mm diameter punch at 0, 2, 4, 6, 8, 12, and 16 days after wounding and either snap-frozen in liquid nitrogen or embedded in optimal cutting temperature (OCT) medium for further use. At indicated time point, wounds were photographed with a digital camera.

### 2.2. Histology and Immunofluorescence

To measure histological characteristics of wounds, each wound was embedded and sectioned through its entirety. For histology analysis, 10 *μ*m cryosections from each wound tissue were fixed in 4% formaldehyde and stained with hematoxylin and eosin. For immunofluorescence analysis, 10 *μ*m cryosections were fixed in ice-cold acetone for 10 minutes and blocked for 1 hour at room temperature. Sections were incubated with anti-*α*-SMA-Cy3 (Sigma C6198) antibody at 4°C, overnight. After 5 min counterstaining with 4′, 6-diamidino-2-phenylindole (DAPI), the slides were mounted and analyzed using Nikon A1RSi Laser Scanning Confocal Microscope.

### 2.3. TUNEL Assay

Double immunofluorescence staining for TUNEL (terminal deoxynucleotidyl transferase-mediated dUTP nick-end labeling) and *α*-SMA was performed to examine apoptotic myofibroblasts in granulation tissues. *α*-SMA staining was initially performed in cryosections according to the protocol described above. Then TUNEL staining was performed using an In Situ Apoptosis Detection Kit (Takara, MK500). After nuclear DNA was probed with DAPI, the slides were mounted and analyzed by Nikon A1RSi Laser Scanning Confocal Microscope. Negative control was obtained by omitting terminal deoxynucleotidyl transferase (TdT) enzyme from the labeling procedure.

### 2.4. Masson's Trichrome Staining

Masson's trichrome staining was performed using a standard staining protocol. In brief, 10 *μ*m frozen sections were fixed with 4% PFA followed by refixation in Bouin's solution. After 10 min in Weigert's iron hematoxyline solution, sections were then dipped in Biebrich scarlet-acid fuchsin solution before differentiating in phosphomolybdic-phosphotungstic acid solution. Following 10 min in aniline blue solution and 5 min rinse in acetic acid solution, sections were dehydrated and coverslipped.

### 2.5. Western Blot Analysis

Skin wounds from GADD34^−/−^ and WT mice were excised using 6 mm biopsy punches and total proteins were dissolved in SDS lysis buffer. Protein concentration was analyzed by BCA Protein Assay Kit (Thermo Scientific). Equal amounts of cell lysates were separated on SDS-PAGE gel. After electrophoresis, the separated proteins were transferred to a nitrocellulose membrane. The membrane was blocked with PBST (0.05% Tween 20) containing 3% nonfat milk for 1 hour and then incubated with the primary antibody at 4°C overnight. Primary antibodies used were Smad2/3 (Cell Signaling 8685), phospho-Smad2/3 (Cell Signaling 8828), GAPDH (Cell Signaling 2118), and caspase-3 (Cell Signaling 9662). The blots were subsequently washed and then incubated with an appropriate horseradish peroxidase-conjugated secondary antibody, goat anti-rabbit IgG (Sigma). Western blots were developed by ECL detection system (GE Healthcare).

### 2.6. Real-Time PCR

For analysis of collagen expression, skin wounds were excised from skin using 6 mm biopsy punches. The wounds and surrounding tissues were homogenized in TRIzol (Invitrogen) and total RNA was extracted according to the manufacturer's instruction. RNA was quantified spectrometrically. Starting from 1 *μ*g RNA, 20 *μ*L cDNA was synthesized using High Capacity cDNA Reverse Transcription Kits (Applied Biosystems). 0.5 *μ*L of each cDNA sample was used to measure mRNA levels. The following primers were used (5′-3′, forward and reverse): Mouse Col1*α*1 (Forward5′-GCTCCTCTTAGGGGCCACT-3′, Reverse5′-CCACGTCTCACCATTGGGG-3′); Mouse Col3*α*1 (Forward5′-CATTCCTCCCACTCCAGACTT-3′, Reverse 5′-CTGTGAATCATGTCCAACTGGT-3′); TGF *β* (Forward5′-GGACTCTCCACCTGCAAGAC-3′, Reverse5′-GACTGGCGAGCCTTAGTTTG-3′); *β*-actin (Forward5′-AGTGTGACGTTGACATCCGT-3′, Reverse5′-GCAGCTCAGTAACAGTCCGC-3′). Expression levels for each gene of interest were calculated by normalizing the quantified mRNA amount to the *β*-actin mRNA. Each sample was assessed in triplicate.

### 2.7. Statistical Analysis

The unpaired two-tailed Student's *t*-test was used to identify statistical significance, multiple comparisons of which were corrected by using the Holm-Sidak method. Data were analyzed by the software GraphPad Prism 6, and a *P* value of <0.05 was considered significant.

The formula of *α*-SMA^+^ area (% = *α*-SMA^+^ area within granulation tissue/granulation tissue area × 100%) was used for calculating the percentage of *α*-SMA^+^ area. The granulation tissue area of each sample was marked by white dotted line according to the hematoxylin and eosin staining of adjacent section. *α*-SMA^+^ area within granulation tissue was quantified from 4 sections by using Image J software.

## 3. Results

### 3.1. Deletion of GADD34 Results in Enhanced Wound Closure

**Figure 1:**
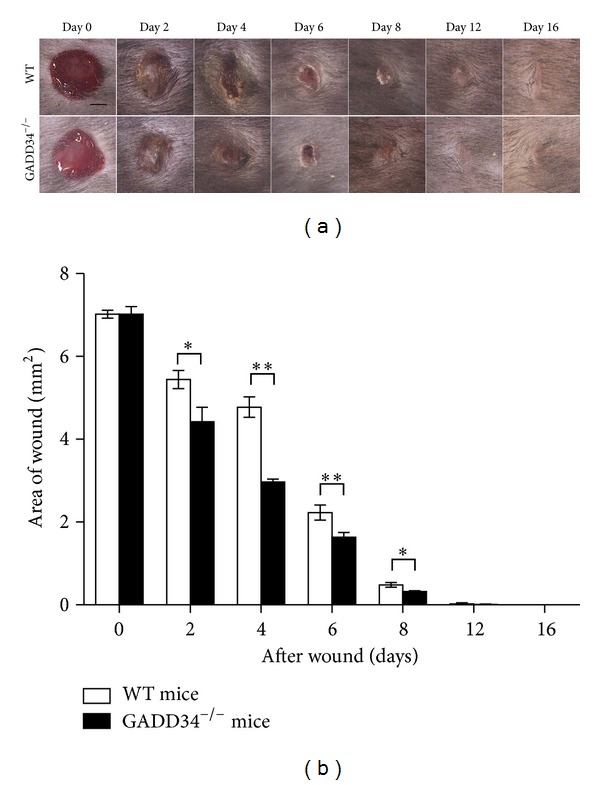
*Loss of GADD34 expression results in enhanced wound closure.* (a) Kinetics of wound closure. Photographs of wounds were captured on days 2, 4, 6, 8, 12, and 16 after wounding to determine the degree of wound closure in GADD34^−/−^ and WT mice. Scale bar: 1000 *μ*m. (b) Diameters of wounds were measured and then the area of each wound was calculated. Representative data shown are means ± SD (day 0: *n* = 4; days 2, 4, 6, 8: *n* = 5; days 12, 16: *n* = 3). *P < 0.05, **P < 0.01.

To investigate whether GADD34-deficient (GADD34^−/−^, KO) mice displayed defects in cutaneous wound closure, we subjected 10-week-old GADD34^−/−^ and WT mice to the dermal punch wound model of wound healing. Wound closure was monitored over a 16-day period and we found that GADD34^−/−^ animals displayed a marked acceleration in wound closure relative to WT mice (Figures 1(a) and 1(b)). On day 2, day 4, day 6, and day 8, the extent of wound closure was significantly greater in GADD34^−/−^ mice than in WT mice.

### 3.2. GADD34 Inhibits Myofibroblasts in Proliferative Phase

**Figure 2:**
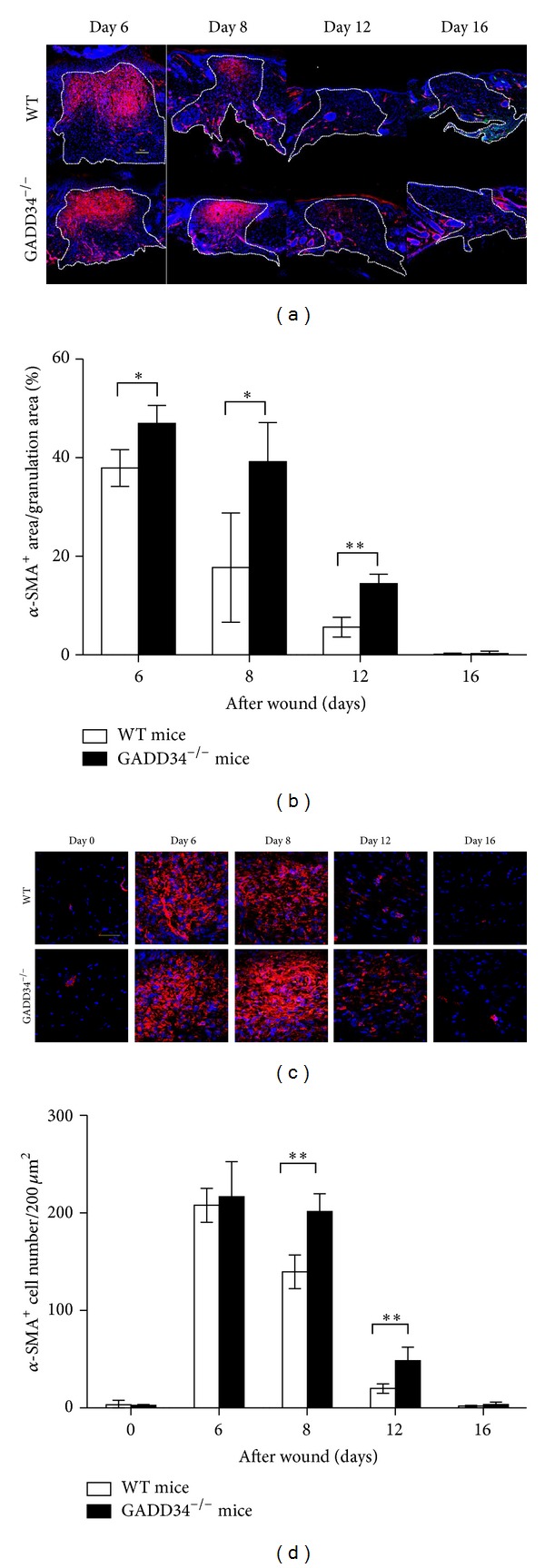
*Myofibroblast differentiation was downregulated by GADD34.α*-SMA expression in granulation tissues from WT and GADD34^−/−^ mice at various time points after injury was analyzed by immunofluorescence assay using antibodies against *α*-SMA (red) and DAPI (blue). (a) Low magnification of wound site of each indicated time point was shown. Selected area by white dotted line means granulation tissue. Scale bar: 100 *μ*m and (b) the percentages of *α*-SMA^+^ area within granulation tissue at each time point were quantified from 4 sections by using image J software. (c) High magnification of wound site was shown. Scale bar: 50 *μ*m and (d) the number of *α*-SMA^+^ cells was counted per 40000 *μ*m^2^ from 4 sections. Bar graph of relative intensity shown is mean ± SD. *P < 0.05, **P < 0.01.

As shown in Figure 1, GADD34^−/−^ mice display significantly increased wound closure compared with WT mice. Due to the vital role of myofibroblasts in wound repair, we measured myofibroblast differentiation by measuring *α*-SMA expression in wound sites of both GADD34^−/−^ and WT mice. Analysis of *α*-SMA expression in wound sites by immunofluorescence revealed that GADD34^−/−^ mice showed elevated *α*-SMA^+^ areas (relative to granulation tissue) 6 (*P* < 0.05), 8 (*P* < 0.05), and 12 days (*P* < 0.001) after wounding compared with WT mice; however, 16 days after wounding, *α*-SMA-expressing myofibroblasts completely disappeared from granulation tissues in both GADD34^−/−^ and WT mice (Figures 2(a) and 2(b)). In Figures 2(a) and 2(b), we did not show day 0's results because there was no granulation formation in normal mouse skin and thus we could not calculate the ratio of *α*-SMA^+^ area to granulation area. The density of *α*-SMA^+^ cells in GADD34^−/−^ mice is higher on day 8 (*P* < 0.001) and day 12 (*P* < 0.01) than WT mice, but not on day 6 (Figures 2(c) and 2(d)). Collectively, the absolute numbers of *α*-SMA-expressing myofibroblasts 6, 8, and 12 days after wounding are higher in GADD34^−/−^ mice than in WT mice, indicating that GADD34 can suppress *α*-SMA-expression in myofibroblasts.

### 3.3. GADD34 Inhibits Collagen Gene Expression during Cutaneous Wound Repair

**Figure 3:**
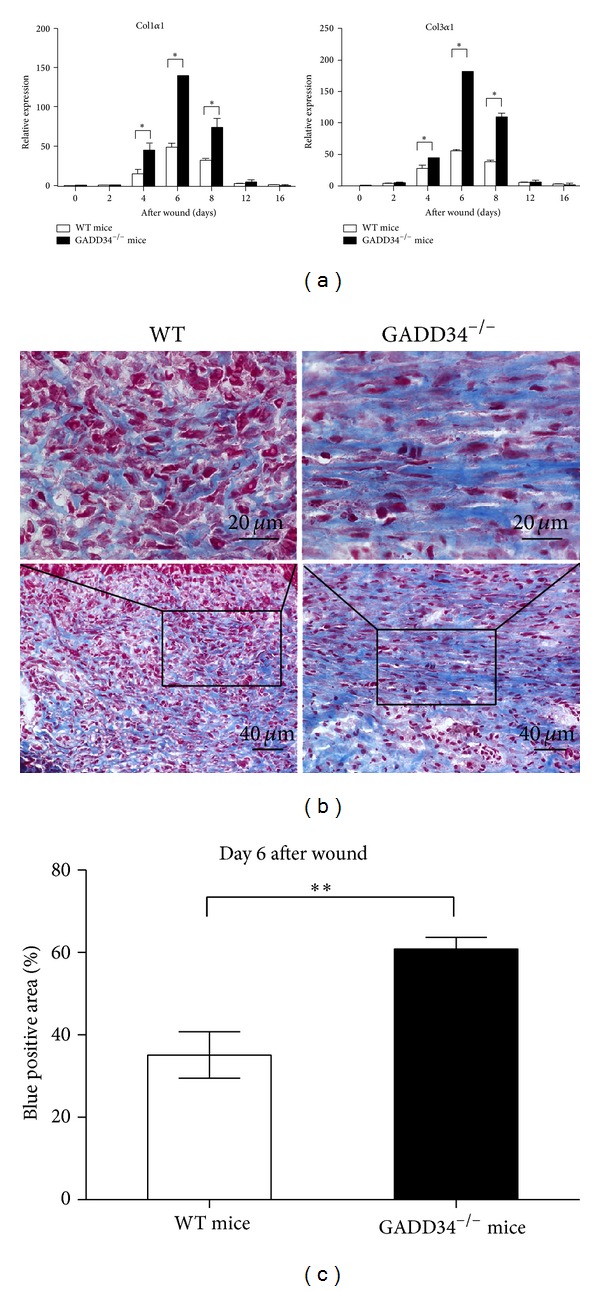
*Enhanced expression of collagen protein during wound healing in GADD34* ^−/−^ *mice.* (a) Relative expression of Col1*α*1 and Col3*α*1 in granulation tissues of WT and GADD34^−/−^ mice from 0 to 16 days after wounding was analyzed by real-time PCR. All results were normalized to *β*-actin values. All experiments were done in triplicate and data presented as means ± SD. (b) Frozen sections from tissues at 6 days after wounding were subjected to Masson trichrome staining. (c) Blue-positive area was calculated from 5 sections using Image J software and data presented as means ± SEM. *n* = 5. *P < 0.05, **P < 0.01.

A key function of myofibroblasts is to produce tension contractile force by secreting extracellular matrix (ECM), particularly, type I and type III collagens [3]. As we observed a marked increase in the number of myofibroblasts in the wounds of GADD34^−/−^ mice, we thought that deposition of collagen should be evaluated more in GADD34^−/−^ mice than in WT mice. So, next, deposition of collagen in wound sites was assessed. Quantitative analysis of wound transcript levels of type I and type III using real-time PCR assay showed enhanced collagen production in days 4, 6, and 8 wound tissues of GADD34^−/−^ mice compared with WT mice. Expression of type I and type III collagens started at day 4 and peaked at day 6, restoring normal level at day 12 in both GADD34^−/−^ and WT mice. During this period, from day 4 to day 8 after wounding, GADD34^−/−^ mice had a 3-fold higher expression of type I and type III collagens than WT mice (Figure 3(a)). To further confirm these findings, Masson's trichrome staining was performed to identify collagen production in the granulation tissue by using day 6's wound tissue (Figure 3(b)), because type I and type III collagens expression is the highest on day 6 according to Figure 3(a)'s results. The results were analyzed by image J software, showing that collagen fibers (blue color) were significantly more common (*P* < 0.01) in GADD34^−/−^ wound granulation tissues (60.84 ± 2.874%), about twofold higher total collagen deposition than WT wound (35.13 ± 5.610%; Figure 3(c)).

### 3.4. GADD34 Suppresses *α*-SMA Expression by TGF *β* Signal Pathway

**Figure 4:**
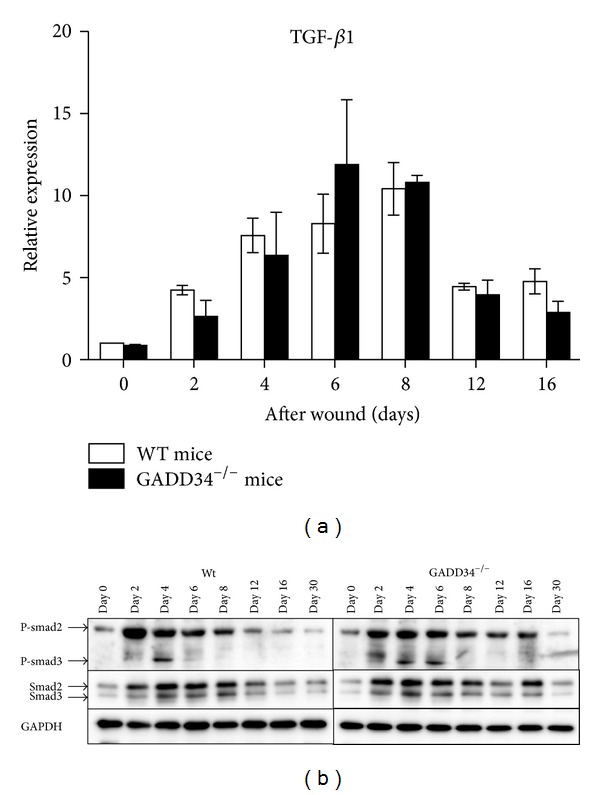
*GADD34 suppresses *α*-SMA expression by TGF *β* signal pathway.* (a) Expressions of mRNA of dermal samples from WT and GADD34^−/−^ mice were determined via real-time PCR. Expression levels are expressed relative to *β*-actin utilizing the ddCt method. Statistics represent 3 independent experiments and results shown are mean ± SD. R-Smad signaling was analyzed by western blot analysis. Cell lysates were prepared from wound site tissues at indicated time point after wounding. Bands were detected with anti-phospho-Smad2/3 antibodies (b). Statistics represent 3 independent experiments.

Transforming growth factor-beta 1 (TGF-*β*1) plays a crucial role in inducing myofibroblast differentiation during wound healing and fibrocontractive diseases through Smad-dependent signaling pathway. So earlier recruitment of myofibroblasts in GADD34^−/−^ mice may be due to higher expression of TGF-*β*1. And we examined the total TGF-*β*1 level of wound site by real-time PCR. However, in contrast to the hypothesis, the data showed that there was no significant difference in TGF-*β*1 mRNA levels between WT and GADD34^−/−^ mice after wounding (*P* > 0.05, Figure 4(a)). Next, we assessed the intracellular effectors of TGF-*β* signaling, the Smad proteins. The phospho-Smad3 in WT mice was expressed at day 4 and returned to normal level at day 6 after wounding, whereas phosphor-Smad3 in GADD34^−/−^ mice was continuously expressed until day 6 after wounding. During that time the expressions of phosphor-Smad2 were almost the same level between WT and GADD34^−/−^ mice (Figure 4(b)).

### 3.5. Deletion of GADD34 Results in Decreased Apoptosis of Myofibroblasts

**Figure 5:**
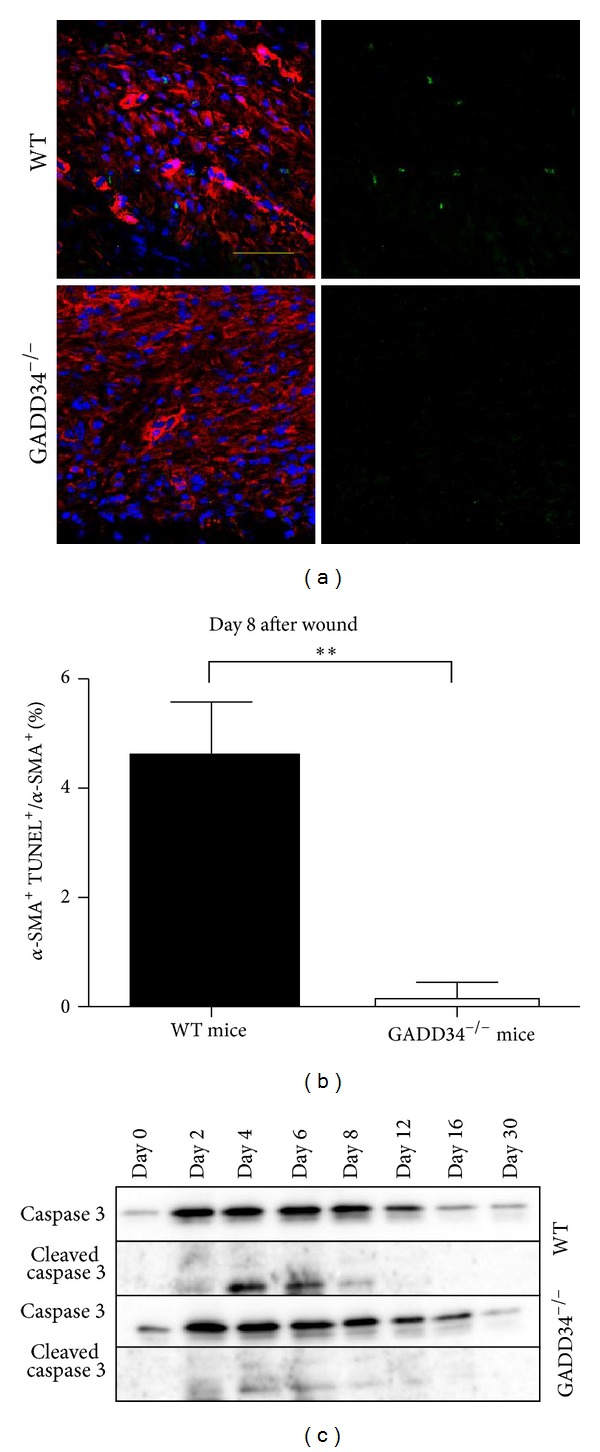
*Induction of myofibroblast apoptosis by GADD34.* (a) Immunostaining of the wound granulation tissues on day 8 was performed using an antibody against *α*-SMA (red), followed by TUNEL staining [27] and DAPI (blue). Scale bar: 100 *μ*m. (b) The percentage of *α*-SMA^+^TUNEL^+^ double positive cells was calculated. Bar graph shown is mean ± SD. **P < 0.01. (c) Immunoblot analyses were performed to assess the levels of cleaved caspase 3 in WT and GADD34^−/−^ mice. Statistics shown represent 3 independent experiments.

As shown in Figure 2, the number of myofibroblasts in WT mice had a significant decrease from day 6 to day 8 after wounding. However, such decrease was not so obvious in GADD34^−/−^ mice. Therefore, we hypothesized that GADD34 may have a function of promoting the apoptosis of myofibroblasts during wound healing. To verify such hypothesis, TUNEL assay and *α*-SMA staining was performed in day 8 wound sections and a significant decrease in the number of apoptotic myofibroblasts (*α*-SMA^+^TUNEL^+^) was observed in GADD34^−/−^ mice (*P* < 0.0001; Figures 5(a) and 5(b)). Correlated with TUNEL assay, marked activation of caspase-3 was detected at day 4, day 6, and day 8 after injuring in WT mice, whereas caspase-3 was weakly activated in GADD34^−/−^ mice (Figure 5(c)). Taken together, these results indicate that GADD34 may promote myofibroblast death through caspase-3-mediated apoptosis during wound repair.

### 3.6. GADD34 Suppresses Hair Follicle Formation during the Remodeling Phase

**Figure 6:**
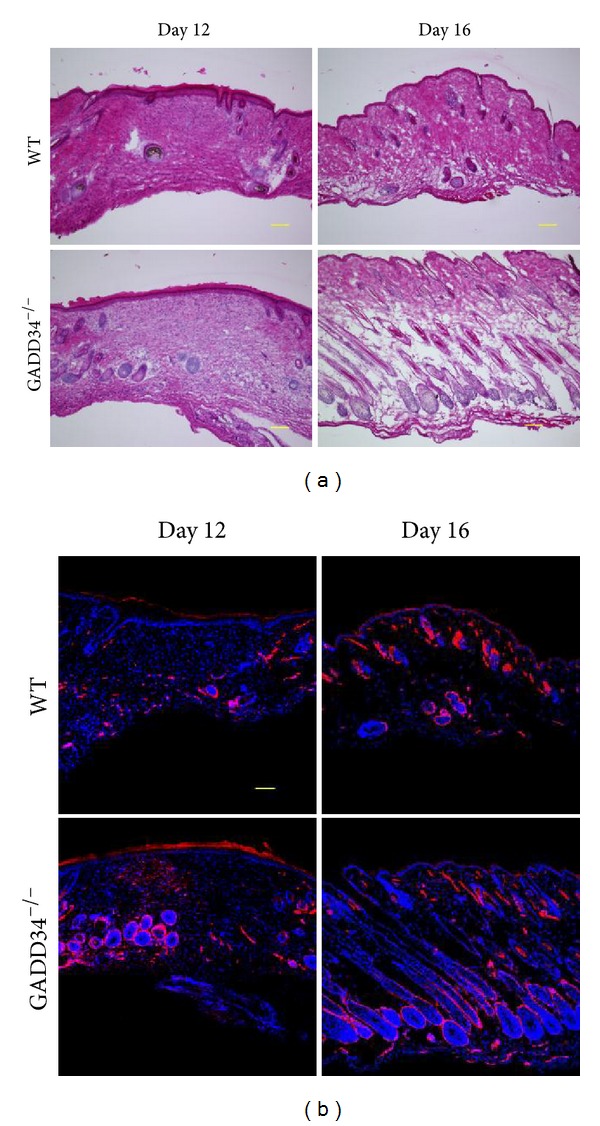
*GADD34 suppresses hair follicle formation at remodeling phase.* Wound granulation tissues of day 12 and day 16 in WT and GADD34^−/−^ mice were subjected to histomorphometric analysis evaluated by H&E staining (a) and immunofluorescent assay by using an antibody against *α*-SMA (red) and DAPI (blue) (b). Data shown represent 3 separate experiments. Scale bar: 100 *μ*m.

When we subjected the wound samples to immunofluorescence assay, much more hair follicle was found to appear in wound site or its surroundings in GADD34^−/−^ mice at day 12 and day 16 (Figures 6(a) and 6(b)) and at day 16 the hair cycle in GADD34^−/−^ mice entered in anagen stage earlier than in WT mice. Combined with previous western blotting results (Figure 4(b)), which showed that phospho-Smad2 level was higher at day 12 and day 16 in GADD34^−/−^ mice, it is indicated that GADD34 might be responsible for suppressing formation of hair follicle through Smad2-dependent TGF*β* signal pathway.

## 4. Discussion

In this paper, we have shown that GADD34 can suppress the myofibroblast differentiation. During cutaneous wound healing, myofibroblasts have three major origins, namely, local tissue connective fibroblasts [19], bone marrow-derived cells, including fibrocytes, a subpopulation of bone marrow-derived leukocytes with fibroblast characteristics [20], and mesenchymal stem cells [21]. However, which types of cells does GADD34 suppress to differentiate into myofibroblasts has not been shown yet. It has been proved that resident fibroblasts are the major source of de novo collagen deposition, whereas BM-derived cells do not contribute to collagen deposition in dermal wound recovery [22]. From the fact that BM-derived cells do not contribute to collagen deposition, we tentatively conclude that GADD34 at least suppresses the differentiation of resident fibroblast to myofibroblast since we observed the significant increase of collagen deposition after wounding in GADD34^−/−^ mice (Figure 3). One of the goals of our future research is to examine which types of cells are suppressed by GADD34 during myofibroblast differentiation.

It is widely accepted that TGF-*β*1 is the major inducer of myofibroblast differentiation, although IL-6 also has been suggested to be such a factor of modulating *α*-SMA expression in dermal fibroblasts [23]. TGF-*β*1 exerts its activity of regulating cell growth, differentiation, and development mainly through Smad-dependent pathway, in which phosphorylation of Smad2 and Smad3 is the key step. So after finding that mRNA level of TGF-*β*1 had no significant change in GADD34^−/−^ mice, we began to examine the phosphorylated levels of Smad2 and Smad3. The results clearly showed that GADD34 could inhibit phosphorylation of Smad3 during myofibroblast differentiation in proliferative phase. Thus GADD34 suppresses *α*-SMA expression via inhibition of Smad3 phosphorylation. When we examined *α*-SMA expression and Smads phosphorylation, we found that more fair follicle formed in GADD34^−/−^ mice and simultaneously Smad2 phosphorylation also increased. Previous investigation has shown that GADD34 can be recruited by Smad7 to dephosphorylate TGF *β* type I receptor which is responsible for activation of Smad2 and Smad3. Thus, through being recruited by Smad7 to inhibit Smad2/3 phosphorylation, GADD34 possibly has a role of universe suppressing activity in other biological processes that are regulated by Smad-dependent TGF *β* signal pathway.

Although GADD34 suppressed collagen synthesis which is the major component of extracellular matrix (ECM) product by myofibroblasts and GADD34 was observed to play a negative role in TGF *β* signal pathway, here we cannot reach a conclusion that GADD34 suppresses collagen synthesis though inhibiting TGF *β* signal pathway. Because there exists a possible mechanism that GADD34 prevents the binding of BFCOL1 to collagen promoter, by which GADD34 may also downregulate collagen accumulation [24]. Thus further work will be necessary to more precisely define the GADD34 function on collagen accumulation during wound healing.

The contribution of GADD34 to apoptotic process remains unclear. The existing reports indicate a double role of GADD34 in cellular apoptosis. The expression of GADD34, caused by both VSV infection [25] and energy depletion [13], can protect cells from apoptotic cell death by activating caspase-3. However, overexpression of GADD34 facilitates cellular apoptosis associated with ionizing radiation [16]. Why there exist conflicting molecular mechanisms has not been elucidated until now. Our data presented here support that GADD34 elicits cellular apoptosis through activating caspase-3, at least during dermal wound repair.

## 5. Conclusion

In summary, we describe the novel finding that GADD34 negatively regulates myofibroblast differentiation on cutaneous wound healing, and we based this conclusion on our results showing that GADD34^−/−^ mice demonstrate increased myofibroblasts and increased production of collagen; in addition, we show that GADD34^−/−^ mice displayed decreased apoptosis. Fibrosis diseases, encompassing a wide spectrum, such as hypertrophic scars, scleroderma, and Dupuytren's disease, even some types of cancer, all of which are characterized by the failure to terminate normal tissue repair, the persistence of myofibroblasts within lesions and a relative resistance to apoptosis, result in unrelenting, progressive ECM secretion and remodeling [3, 26]. So, how to inhibit myofibroblast differentiation becomes one of the important considerations of treating such type of diseases. Our studies therefore provide a novel perspective to suppress myofibroblasts in different stages, which may have long-term therapeutic implications for the treatment of both homeostatic and pathological wound healing processes.
